# First case report of pancreatic angiomyolipoma diagnosed by EUS-guided fine-needle biopsy

**DOI:** 10.1097/eus.0000000000000065

**Published:** 2024-07-16

**Authors:** Francisco Vara-Luiz, Marta Patita, Pedro Pinto-Marques, Ivo Mendes, Ana Ramos Canastra

**Affiliations:** 1 Gastroenterology Department, Hospital Garcia de Orta, Almada, Portugal; 2 Egas Moniz Center for Interdisciplinary Research (CiiEM), Egas Moniz School of Health and Science, Caparica, Portugal; 3 Gastroenterology Department, Hospital CUF Tejo, Lisboa, Portugal; 4 Pathology Department, Hospital CUF Descobertas, Lisboa, Portugal.

**Figure 1:**
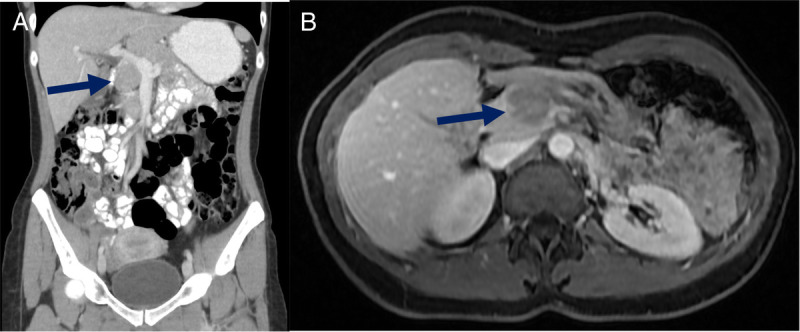
Computed tomography (A)/magnetic resonance (B) showing a hypodense/hypointense hypovascular nodule in the pancreatic head.

**Figure 2:**
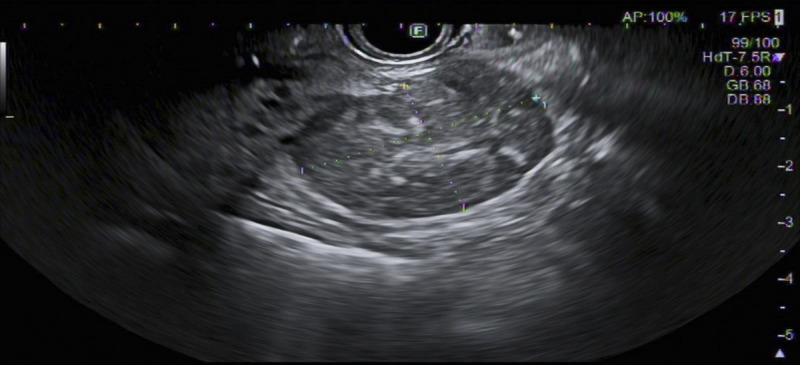
EUS showing a 33 × 23-mm heterogenous mass in the transition head/istmus, without vascular contact or wirsung dilation.

**Figure 3:**
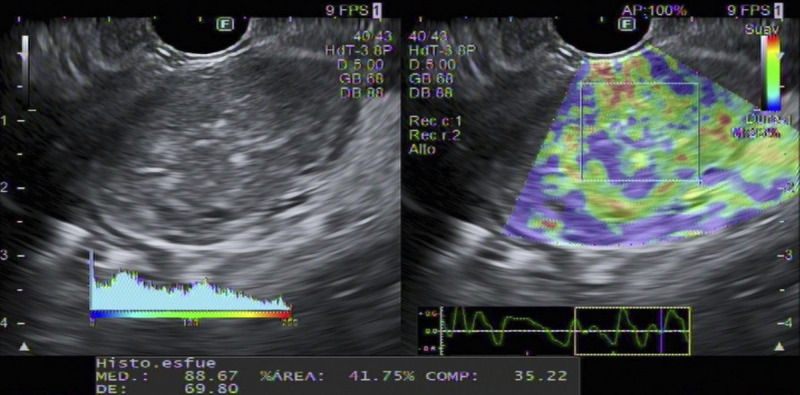
Quantitative elastography evaluation showed strain histogram (SR) 89.

**Figure 4:**
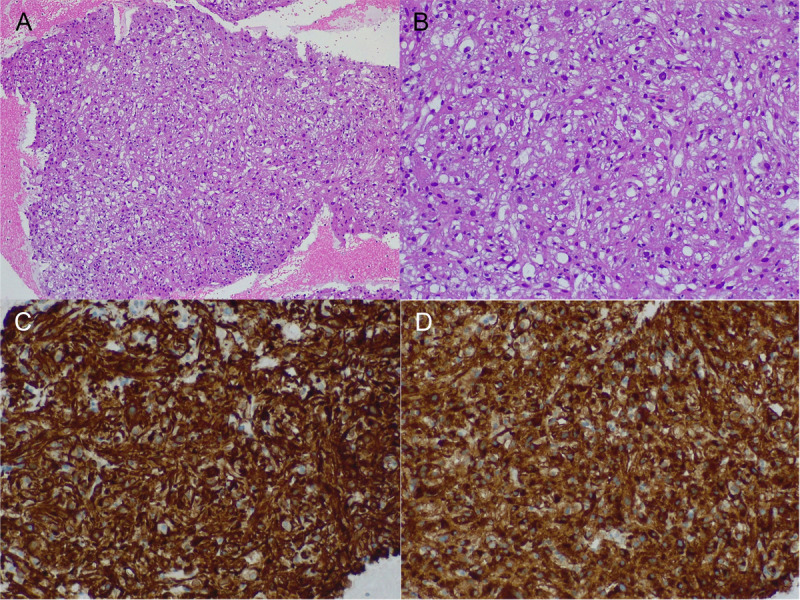
Histopathology examination showing mature adipose tissue, smooth muscle cells and thick-walled blood vessels (A,B). Immunohistochemistry for smooth muscle (C) and melanocytic (D; HBM-45) markers and were compatible with pancreatic angiomyolipoma.

A 37-year-old female with unremarkable history underwent routine abdominal ultrasound, which revealed a heterogenous pancreatic mass. Further evaluation with computed tomography and magnetic resonance imaging showed a 28 × 26-mm slightly hypodense/hypointense, hypovascular nodule in the pancreatic head [Figure 1]. Laboratory examination was within the normal range, including carbohydrate antigen 19-9 (CA19-9)/carcinoembryonic antigen (CEA). The EUS revealed a 33 × 23-mm heterogenous mass in the transition head/istmus, without vascular contact or wirsung dilation [Figure 2]. Quantitative elastography showed strain histogram 89 [Figure 3]. The fine-needle biopsy (FNB) using a 19G needle revealed a mesenchymal neoplasm composed of mature adipose tissue, smooth muscle cells, and thick-walled blood vessels [Figure 4]. Immunohistochemistry for melanocytic (HBM-45) and smooth muscle (desmin) markers were strongly positive, whereas no immunoreactivity was seen with epithelial markers, compatible with pancreatic angiomyolipoma (AML).

AML is a mesenchymal tumor that most commonly affect the kidney. Because bleeding and rupture are known complications of renal AML, the same can be expected from extrarenal tumors. Surgery is the treatment of choice, allowing disease cure.^[1,2]^ To our knowledge, there are only 4 cases in the literature of primary pancreatic AML,^[1–4]^ and this is the first diagnosed by EUS-FNB. We highlight pancreatic angiomyolipoma as a rare entity in the differential diagnosis of a pancreatic mass.
